# Performance improvements from imagery: evidence that internal visual imagery is superior to external visual imagery for slalom performance

**DOI:** 10.3389/fnhum.2013.00697

**Published:** 2013-10-21

**Authors:** Nichola Callow, Ross Roberts, Lew Hardy, Dan Jiang, Martin Gareth Edwards

**Affiliations:** ^1^School of Sport, Health and Exercise Sciences, Institute for the Psychology of Elite Performance, Bangor UniversityBangor, UK; ^2^Institute of Psychological Sciences and Institute of Neuroscience, Université catholique de LouvainLouvain-la-Neuve, Belgium

**Keywords:** mental practice, kinesthetic imagery, imagery ability, VMIQ-2, speed-accuracy tradeoff

## Abstract

We report three experiments investigating the hypothesis that use of internal visual imagery (IVI) would be superior to external visual imagery (EVI) for the performance of different slalom-based motor tasks. In Experiment 1, three groups of participants (IVI, EVI, and a control group) performed a driving-simulation slalom task. The IVI group achieved significantly quicker lap times than EVI and the control group. In Experiment 2, participants performed a downhill running slalom task under both IVI and EVI conditions. Performance was again quickest in the IVI compared to EVI condition, with no differences in accuracy. Experiment 3 used the same group design as Experiment 1, but with participants performing a downhill ski-slalom task. Results revealed the IVI group to be significantly more accurate than the control group, with no significant differences in time taken to complete the task. These results support the beneficial effects of IVI for slalom-based tasks, and significantly advances our knowledge related to the differential effects of visual imagery perspectives on motor performance.

## Introduction

Research examining the effects of imagery on the acquisition and execution of motor performance has delineated imagery into modalities and perspectives. This delineation includes visual and kinesthetic sensory modalities (e.g., Hardy and Callow, 1999; Fourkas et al., 2006; Guillot et al., 2009), with the visual modality being further separated into two visual imagery perspectives. These two perspectives are: internal visual imagery perspective (IVI: where the imaginer is looking out through his or her own eyes while performing the action) and external visual imagery perspective (EVI: where the imaginer is watching him or herself performing the action from an observer's position; as if watching him or herself on television). Refer to Callow and Roberts (2012) for further detail surrounding visual imagery perspective conceptualization. The kinesthetic imagery modality is defined as how it feels to perform an action, and includes aspects such as the force and effort involved in movement (Callow and Waters, 2005).

Early research exploring the effect of internal and external visual imagery produced equivocal results. For example, Mahoney and Avener (1977) revealed that successful qualifiers for the U.S. Olympic gymnastics team used internal imagery more than non-qualifiers. However, in contrast to this, Ungerleider and Golding (1991) found that successful U.S. track and field athletes used more external imagery than non-successful athletes. In addition, some experimental studies (e.g., Epstein, 1980) found no differences between imagery perspectives and their effects on performance. Three possible explanations have been provided for these inconsistent results: (a) that specific visual imagery perspectives produce greater performance gains for certain motor tasks than for others (e.g., Highlen and Bennett, 1979; Hardy, 1997), (b) that previous conceptualizations of internal imagery (such as that used by Epstein and Mahoney and Avener) have confounded internal visual imagery with kinesthetic imagery (cf. Hardy and Callow, 1999), and (c) that it has been incorrectly assumed that kinesthetic imagery can only be experienced with an internal perspective or is easier to use with an internal perspective (cf. White and Hardy, 1995; Taktek, 2012).

Hardy and associates (White and Hardy, 1995; Hardy, 1997; Hardy and Callow, 1999) examined the task specificity explanation (part “a” above), and offered two hypotheses to explain the effects of different visual imagery perspectives on different motor tasks. In the first (EVI) hypothesis, they posited that EVI would be superior to IVI for tasks that require positioning the body relative to itself, such as tasks relying heavily on the use of “form.” To test this hypothesis, White and Hardy (1995) examined the performance of a simulated rhythmic gymnastics routine following the use of either EVI or IVI. Results revealed that the EVI group made fewer accuracy errors in performance than the IVI group. Hardy and Callow (1999) confirmed this finding with a series of three ecologically valid tasks relying heavily upon the use of form for their successful completion. In all three tasks, (i.e., a karate kata task, a gymnastics floor routine, and a technical rock climbing task), the use of EVI was found to have a superior influence on performance compared to the use of IVI. Taken together these results provide support for Hardy's (1997) cognitive explanation for the EVI hypothesis. Specifically, Hardy suggested that imagery exerts a beneficial effect on performance only to the extent that the images generated supplement the information that is already available to the performer. Thus, for tasks relying heavily upon the use of form, EVI may be more useful than IVI because EVI would allow a performer to see the desired form associated with the correct movement.

In the second (IVI) hypothesis, Hardy and associates suggested that IVI would be superior to EVI for tasks that require positioning the body in relation to other external visual features, such as in slalom-based tasks where a performer has to follow a “line” through or around a set course (e.g., downhill slalom skiing), with the cognitive explanation (Hardy, 1997) that IVI may allow a performer to see the precise temporal and spatial locations where key movements need to be initiated (e.g., changing direction or “braking”) from the actual viewing angle of the motor action in relation to external visual information. Thus, the temporal and spatial locations would be identified with reference to the performer's position on the actual line being taken, which would afford critical visuomotor information that would not be available or useful when using EVI.

To test the second hypothesis, White and Hardy (1995) used a wheelchair slalom task that required the participant to maneuver themselves through a set course of gates. The results showed that after initial practice on an acquisition course, participants using IVI completed a transfer trial with significantly fewer accuracy errors than participants using EVI. Therefore, use of IVI compared to EVI led to a more accurate performance, explained by participants being able to rehearse the responses required at each gate. However, the results also showed that EVI improved the speed at which the task was performed compared to IVI. White and Hardy suggested that these performance gains occurs because EVI allows participants to compare themselves with their own imagery, thereby enhancing their competitive drive. Following this line of reasoning, as IVI does not afford the comparison to the same extent as EVI, the motivation function is perhaps less evident for IVI. White and Hardy further discussed these findings in terms of a speed accuracy trade off *across* imagery perspectives, where IVI caused slow, but accurate performance and EVI caused a fast, but inaccurate performance.

More recently, a number of neuroimaging studies have shown differences in neural activity dependent on the imagery perspective taken (e.g., Ruby and Decety, 2001; Fourkas et al., 2006; Lorey et al., 2009). These neural differences have then been used to explain the differential effects of imagery perspectives on performance, via the notion of functional equivalence (e.g., Jeannerod, 1994, 2001; Hanakawa et al., 2008). That is, the more similar (functionally equivalent) the imagery of performance and the actual performance is, the more effective the imagery is at moderating performance (cf. Holmes and Collins, 2001; Smith et al., 2008; Wakefield et al., 2013). However, the conceptualization of imagery perspectives used in the neuroimaging studies differ markedly to both our conceptualizations of IVI and EVI, and the current received view in the sport psychology literature (e.g., Cumming and Ramsey, 2008; Moran, 2009; Tobin and Hall, 2012). For example, neuroscientific conceptualizations of internal imagery confound visual and kinesthetic modalities (e.g., Ruby and Decety, 2001; Lorey et al., 2009), and external imagery is usually of someone else (e.g., Ruby and Decety, 2001; Fourkas et al., 2006; Lorey et al., 2009). While several other fMRI (e.g., Guillot et al., 2009) and psychophysiological studies (e.g., Guillot et al., 2004) are clear to make distinctions between imagery modalities (i.e., visual and kinesthetic), these studies do not examine visual perspective differences. Consequently, a precise understanding of what neural areas are involved in internal visual imagery and external visual imagery is not known, and, thus the current neuroscientific research cannot be used to precisely explain the differential effects of *visual* imagery perspectives on performance. Having said this, we might assume similar neural functional equivalence between the specific visual imagery perspectives and the different tasks, with a slalom-based task being particularly moderated by internal visual imagery, or a form-based task being particularly moderated by external visual imagery (i.e., demonstrating specific functional equivalence; cf. Callow and Roberts, 2010).

The imagery perspective behavioral research literature already supports Hardy and associates EVI hypothesis, but support for the IVI hypothesis is not yet conclusive. In the present research we examined the IVI hypothesis by exploring the effects of IVI and EVI on three different slalom tasks; a driving-simulation slalom task in Experiment 1, a downhill slalom running task in Experiment 2, and a downhill ski-slalom task in Experiment 3. Although these tasks differ in their specific requirements, they all require a performer to follow a “line” through or around a set course in order to gain a fast performance time. We therefore suggest that IVI would be expected to be particularly beneficial in moderating performance for these tasks.

## Experiment 1: driving-simulation slalom task

### Methods

#### Participants

A sample of 45 male participants was recruited (*M* age 21.35 = years *SD* = 3.12). The participants were all right-handed and had normal or corrected-to-normal vision. All participants held a UK driving licence for a minimum of 1 year, but reported that they had never played the specified driving game used in the experiment, and played computer games on average less than once per week in the preceding 6 months. All participants provided written informed consent, and ethical approval for the experiment was granted by the School's Ethics Board.

#### Experimental apparatus and task

The driving-simulation slalom task was undertaken in a purpose-built driving simulator, incorporating a rally car seat, a force feedback steering wheel (which could be turned ±900° to keep the car on the circuit), 6-speed gear shifter and pedals. The driving simulator was connected to a 22 inch LCD monitor displaying the Gran Turismo 5 Prologue game (Codemaster, Warwickshire). In a training phase of the experiment, the track used was the Suzuka Circuit, which was 3.61 miles long and consisted of 20 bends (nine left and 11 right). In the experimental phase, the chosen track was the Eiger Nordward circuit, which is 1.51 miles long and consists of 11 bends (five left and six right). In both phases, the circuits were driven as a time trial in dry, daylight conditions, with a Citroen C4 2.0 VTS Coupe'05 as the test car. The virtual reality display presented the driver's view out through the front window of the car as if actually driving the car.

#### Experimental phases

In order to train the participants to use the apparatus for the experimental phase, participants completed a 90-min training phase period where they had to achieve two criteria (derived from pilot testing). This included the completion of three consecutive laps under 170 s and a plateau in performance, where the last three lap times fell within 5 s of each other (cf. Wilson et al., 2007). If participants achieved the criteria, they then proceeded to the experimental phase. In the experimental phase, participants completed a total of 15 laps (five practice, five pre-imagery, five post-imagery) of the simulated rally driving circuit, with average lap time at pre and post-imagery condition used as the measure of change in performance. The participants were randomly assigned to one of three groups; internal visual imagery (IVI), external visual imagery (EVI), or maths-control. Following practice and pre-imagery performance measures, participants in the imagery groups were given an imagery script pertaining to the imagery group to which they were allocated. The IVI script detailed the task from a first person visual perspective, requiring the participants to image the task through their own eyes. The EVI script detailed the task from the perspective of a third person visual perspective, requiring the participants to see themselves performing the task. All scripts were developed using Lang's (1984) guidelines for including stimulus, response and meaning propositions into the script, and pilot tested (and amended based on feedback) prior to data collection. In order to maintain experimental control, the scripts were developed by the authors. However, there was flexibility in the scripts (e.g., participants in the IVI group were asked to imagine their view change as they turned a corner). This flexibility allows participants to develop their own images, thus providing a degree of individualization, and consequently the images being meaningful for the participants (cf. Wilson et al., 2010). The scripts took ~120 s to administer. Example excerpts from the scripts are as follows:

(Example 1, IVI.) Crossing the start line, you see the long straight in front of the car. Notice as the front of the car is going downhill slightly; it is traveling over a couple of horizons. As you approach the S-shape bend head, you see the line you want to take. As the car approaches the bend, you break to take the perfect line, turning first to your right and then quickly to your left, staying close to the bend, and accelerating after the bend.
(Example 2, EVI.) As the car crosses the start line, see the long straight in front of it. Notice that the car is going downhill slightly and is traveling over a couple of little horizons. As you see the car approach the S-shape bend ahead, you see the line you want it to take. As the car approaches the bend, you see yourself allowing the car to break to take the perfect line, seeing yourself turn the wheel first to your right and then to your left, staying close to the bend, and accelerating after the bend.

In the control condition, participants were required to answer standard arithmetic questions (e.g., 14 + 4 + 6). This type of active control group has been demonstrated to prevent the use of imagery during the experiment, but does not interfere with performance on the dependent variable (cf. Driskell et al., 1994; Callow and Hardy, 2005).

#### Measures

Time-taken to complete each lap was measured automatically (in seconds) by the Gran Turismo 5 Prologue software, and recorded by the experimenter. Note that the line of driving moderated the time, with cutting corners reducing the time compared to driving in the center of the road, but with collisions with curbs, or driving on the grass adding to the time. In order to determine participants' imagery ability, all participants completed the Vividness of Movement Imagery Questionnaire-2 (VMIQ-2: Roberts et al., 2008). The VMIQ-2 has demonstrated acceptable factorial validity, construct validity and concurrent validity (see Roberts et al., 2008). The VMIQ-2 comprises 12 items that assess the ability to image a variety of movements. Participants are required to image each item using IVI, EVI, and kinesthetic (Kin) imagery, and rate the vividness of the image produced on a five-point Likert scale from 1 (*perfectly clear and vivid*) to 5 (*no image at all*). Cronbach's alphas for the current study were 0.86 (EVI), 0.90 (IVI), and 0.84 (Kin). A manipulation/social validation questionnaire was also administered. The first question, asked all participants whether they had been able to adhere to the treatment group. The remaining questions were only given to participants in the two visual imagery groups, and they were asked whether they had experienced any switching of visual imagery perspectives during the task, and whether they had experienced any kinesthetic imagery during their use of visual imagery. An 11-point Likert scale ranging from 0 (*not at all*) to 10 (*very much so*) was employed.

### Procedure

One week prior to the commencement of the experiment, participants completed the VMIQ-2. All participants achieved a criterion of equal to or less than 36 on each of the VMIQ-2 subscales, indicating that their imagery ability was *at least moderately clear and vivid*. Participants attended the laboratory individually and were instructed that the purpose of the experiment was to examine driving ability under different conditions. The experimenter read standardized instructions detailing the training and experimental phases to the participants. Participants then completed the 90 min training phase, and all participants achieved the criterion level. On completion of the training phase, participants were given a 15 min break before commencing the experimental phase. The experimenter read standardized instructions explaining that they were to complete a number of trials as fast as they could; five practice trials, then five pre-imagery test trials and then five post-imagery test trials^1^. Before each of the post-imagery test trials, participants in the IVI and EVI groups listened to a recording of the imagery script detailing the driving task from the visual imagery perspective to which they were assigned, and were asked to use the imagery prior to performing each of the trials. Participants in the control group solved 10 maths questions prior to each post-test trial, as pilot testing had revealed that the calculation of 10 maths questions equated to the average time taken to complete the imagery scripts. Upon completion of each post-imagery test trial, participants rated the extent to which they drove as fast as they possibly could on an 11 point Likert scale from 0 (*not at all*) to 10 (*very much so*), with the intent that any participant who scored less than 5 would be asked to repeat the trial. In the event, no participants scored less than 5 for any trial. At the end of the post-imagery test trials, participants completed the manipulation/social validation questionnaire. On completion of the questionnaire, the participants were de-briefed as to the nature of the experiment and thanked for their participation.

### Results

#### Preliminary analyses

All participants reported on the manipulation/social validation questionnaire that they were able to adhere to their allocated groups with minimum reported switching of perspectives in either of the imagery groups (i.e., a score of less than 3 was used at the cut-off criteria indicated that participants rarely, if at all, switched between IVI and EVI when they were only supposed to be using one perspective). Therefore, no participants were removed from the analysis. Participants in the IVI and EVI groups reported some experience of kinesthetic imagery during their visual imagery (see Table 1 for descriptive results), although there was no significant difference between the imagery groups in terms of their kinesthetic imagery experience (*p* = 1, *d* = 0). Analysis of the VMIQ-2 data (using a bonferroni adjusted α of 0.017) revealed no differences between the different participant groups for IVI imagery ability *F*_(2, 42)_ = 0.42, *p* = 0.66, η^2^ = 0.02 1-β = 0.11, and kinesthetic imagery ability *F*_(2, 42)_ = 1.32, *p* = 0.28, η^2^ = 0.01 1-β = 0.27. However, for EVI imagery ability, there was a significant difference between the groups *F*_(2, 42)_ = 7.48, *p* = 0.002, η^2^ = 0.26 1-β = 0.93, with the EVI group showing significantly better EVI ability than the IVI group (*p* = 0.003, *d* = 1.66) and the control group (*p* = 0.009, *d* = 1.45).

**Table 1:** **Kinesthetic experience and lap-time (seconds) at pre-test and post-test in Experiment 1**.

**Group**	**Kinesthetic experience *M* (*SD*)**	**Pre-test lap time *M* (*SD*)**	**Post-test lap time *M* (*SD*)**
IVI	4.43 (3.00)	88.08 (2.10)	86.23 (1.78)
EVI	4.53 (3.02)	87.55 (1.94)	87.45 (1.92)
Control	–	87.67 (2.10)	87.57 (2.41)

#### Performance score (time-taken)

A mixed-model (group × test) ANOVA was employed to analyse the average lap-time at pre and post-test. Box's *M* test for homogeneity of dispersion matrices was significant. Data transformations failed to rectify this problem. However, Stevens (2002) states that if Box's *M* test is significant with approximately equal numbers in each group, the Type I error rate will only be slightly affected, whereas power will be weakened. Thus, it remains relatively safe to interpret significant effects, because they are robust enough to show significance despite the low power. Consequently, the results from the analysis on the raw (non transformed) data are reported here. The analyses revealed no significant main effect for group, *F*_(2, 42)_ = 0.23, *p* = 0.80, η^2^ = 0.01 1-β = 0.08, a significant main effect for test *F*_(1, 42)_ = 18.57, *p* < 0.001, η^2^ = 0.21, 1-β = 0.99 and a significant group by test interaction, *F*_(2, 42)_ = 13.65, *p* < 0.001, η^2^ = 0.31, 1-β = 0.99. Tukey's tests on the significant interaction revealed that there was no significant difference between groups at pre-imagery tests. However, at post-imagery tests, the internal visual imagery group performed significantly better than the external visual imagery group *q*_(42)_ = 6.31, *p* < 0.05, *d* = 0.66 and the control group *q*_(42)_ = 6.94, *p* < 0.05, *d* = 0.63. In addition, the IVI group significantly improved performance from pre to post-test *q*_(14)_ = 9.56, *p* < 0.05, *d* = 0.98. No other differences were significant. See Table 1 for descriptive results.

Given that kinesthetic imagery can cause performance gains over and above those caused by visual imagery (Hardy and Callow, 1999), it was important to establish if the kinesthetic imagery used in the two conditions could have influenced the results (despite there being no significant differences in the experience of kinesthetic imagery in the two visual imagery groups). We examined the relationship between kinesthetic imagery and performance and found no significant correlation between kinesthetic imagery (reported from the manipulation/social validation questionnaire) and performance (average lap-time) at post-imagery test (*r*_*s*_ = 0.06, *p* = 0.77). Thus, the superior performance for the IVI group could not be attributed to differences in kinesthetic imagery experience between the two groups.

### Discussion

The results of this first experiment offer clear support for the hypothesis that internal visual imagery appears superior to external visual imagery in a driving-simulation slalom task. Further, although there was no relationship between kinesthetic imagery and performance, contrary to previous debate that kinesthetic imagery cannot be used with external visual imagery (cf. Collins and Hale, 1997) the results provide further evidence that kinesthetic imagery can be experienced with both internal visual and external visual imagery. The theoretical and applied implications of the findings are discussed later in the General discussion section.

## Experiment 2: downhill slalom running task

Although Experiment 1 used methods and procedures that afforded substantial experimental control, the participants were not actually moving through the visual field while performing the task, as they would do in sports such as canoe slalom and slalom skiing. It therefore could be argued that the findings of Experiment 1 alone lack in ecological validity. In Experiment 2, we aimed to replicate and extend Experiment 1 by using a more ecologically valid task. This involved downhill slalom running where participants actually moved through the visual field while completing the task. In addition, the task allowed for separate measures of time taken (as in Experiment 1) and accuracy (as reported in White and Hardy, 1995).

We *a* priori hypothesized that the use of IVI would produce superior performance in comparison to EVI. This hypothesis was partly based on the results of Experiment 1, and also based on two further arguments. Firstly, IVI involves the rehearsal of precise changes in direction at particular spatial positions (for example, the angle at which to change direction to move past a cone or object), helping participants to plan and select the best line. We propose that this rehearsal may help the participants to better plan and execute the slalom line during the task performance.

The second reason for expecting IVI to produce superior performance compared to both EVI was due to task differences between the current task of downhill slalom running and the wheelchair slalom task used by White and Hardy (1995). More specifically, as White and Hardy's wheelchair slalom task relied heavily on the *generation* of speed (because participants performed the task on a flat surface), the motivational function of EVI might have reduced the time taken, via enhanced competitive drive. In contrast, downhill slalom running relies more heavily on the *control* of speed (due to the effect of momentum) rather than speed generation. Thus, the motivational function of EVI would likely be less relevant.

### Methods

#### Participants

A sample of 22 sports science students (*M* age = 22.50 years, *SD* = 3.08; 18 men, four women) was recruited for the experiment. All participants provided written informed consent, and ethical approval for the experiment was granted by the School's Ethics Board.

#### Tasks and experimental conditions

The experiment consisted of a practice and an experimental task. In both tasks participants completed a downhill slalom running course, performance was measured in terms of time-taken and the frequency of cone touches (accuracy). The practice and experimental slalom courses were performed outdoors on a disused road that sloped downhill at an angle of 5°. Both courses were 55 meters in length and used 13 cones and automatic timing gates placed at the top and bottom of the course. The actual set-up of the courses differed substantially. On the practice course, the cones were placed at reasonably long intervals, allowing participants to make wider (and less extreme) turns. In contrast, the experimental course required the participants to make more extreme changes in direction. Cones were placed much closer together, and on narrower angles, requiring “tighter” turns in order to be able to maintain a good line through the cones and to be able to complete the task quickly.

A repeated measures design was employed whereby participants completed the task in IVI and EVI conditions. The conditions were completed over two consecutive days and the order was counterbalanced across participants. In each condition, participants were administered a recorded imagery script that corresponded to their condition prior to completing the experimental task trials^2^. Scripts were developed using the same procedures and principles used in Experiment 1.

#### Measures

Using automatic timing gates placed at the top and bottom of the course, the time taken (in seconds) to complete each trial was recorded. To measure accuracy, an independent judge visually observed and then recorded the frequency of cone touches by participants for each trial. As with Experiment 1, imagery ability was measured using the VMIQ-2, and a manipulation/social validation questionnaire administered to assess adherence to the conditions, switching of visual imagery perspectives, and experience of kinesthetic imagery during the use of visual imagery.

### Procedure

The VMIQ-2 was administered 1 week prior to the commencement of the experiment, and all participants achieved the criteria used previously in Experiment 1. Participants were tested individually and on arriving at the experimental site, were equipped with wrist and hand protectors and sports clothing to cover all of the body. The equipment served as protection in case any participants fell while running. Participants were told that the purpose of the experiment was to examine the effects of different imagery scripts on a motor performance task. Standardized instructions were read informing the participants to complete the task as quickly and accurately as possible. Participants performed five practice trials on the practice course and were given a 3 min rest between trials. At the end of the practice trials, participants were given a 5 min break. Participants then entered the experimental phase of the experiment. Prior to performing the first experimental trial, participants were read the same standardized instructions as before, and were allowed to look at the new course and walk down the side of it. Participants were administered the relevant imagery script and were asked to employ imagery before completing each of two experimental trials. Only two experimental trials were employed (compared to five in Experiment 1), because this is the number of competitive trials that are performed in sports such as Super G in alpine downhill skiing and canoe slalom. At the end of each experimental trial, to ensure that the participants complied with the instructions, participants were asked to rate to what extent they ran as fast as possible down the course, on an 11 point Likert scale from 0 (*not at all*) to 10 (*very much so*). As with Experiment 1, no participants scored below five. On completion of the second experimental trial, a manipulation/social validation questionnaire was completed. The questionnaire examined the extent to which participants adhered to their treatment conditions, the experience of kinesthetic imagery, and the extent to which they switched between imagery perspectives. These questions were scored on the same Likert scale used in Experiment 1.

The participants returned the following day and performed the exact same procedure; though used the other imagery perspective. In the second day, following completion of the manipulation/social validation questionnaire, the participants were de-briefed as to the nature of the experiment and thanked for their participation.

### Results

#### Preliminary analyses

To control for potential carryover effects as a result of the repeated measures design, a strict exclusion criteria was employed (Stevens, 2002). Participants were only retained for analysis if they were able to meet two criteria from the manipulation/social validation questionnaire. First, participants were required to be able to report strong adherence to both the IVI and EVI conditions (as evidenced by adherence ratings of seven out of 10 or above for each imagery condition). Second, participants had to report minimal switching between imagery perspectives during each imagery condition (i.e., a score of less than 3 for each condition which would indicate that participants rarely, if at all, switched between IVI and EVI during a particular imagery condition when they were only supposed to be using one perspective). These criteria resulted in the data from 11 of the 22 participants being retained for analysis. Analyses showed that there was no difference in imagery ability across perspectives for these participants, *t*_(10)_ = 1.06, *p* = 0.31, *d* = 0.45.

#### Performance data

A dependent *t*-test was employed to examine the effects of the visual imagery perspective in the experimental trial that the participants ran the fastest in. Results revealed a significant difference for condition, *t*_(10)_ = −3.29, *p* < 0.008, *d* = 0.42. Inspection of the cell means revealed that the course was completed significantly quicker in the IVI condition than in the EVI condition (See Table 2 for descriptive results). A statistical analysis of accuracy was not possible as none of the participants touched a cone in either of the conditions.

**Table 2:** **Kinesthetic experience and time taken (seconds) in Experiment 2**.

**Condition**	**Kinesthetic experience *M* (*SD*)**	**Time taken *M* (*SD*)**
IVI	6.18 (3.19)	15.19 (1.15)
EVI	5.10 (2.54)	15.70 (1.24)

The results support the hypothesis, showing that IVI produced performance gains over EVI. This performance effect was shown via a reduction in time taken. In addition, there was no detriment to accuracy that is participants did not compromise accuracy, at least by colliding with the cones on spatially close turns, in order to achieve the lower times. This leads to the interpretation that the effect for time taken for IVI was not a result of a speed-accuracy trade-off across imagery perspectives. Further to this, and in line with Experiment 1, we wanted to confirm that the quicker times were not a result of greater kinesthetic imagery experience in the IVI condition. Participants reported (via the social validation/manipulation questionnaire) that they experienced kinesthetic imagery in both IVI and EVI conditions, although there was no significant difference between kinesthetic imagery experience in the two conditions (*p* = 0.14, *d* = 0.38). Further the experience of kinesthetic imagery was uncorrelated with performance (*r*_*s*_ = 0.006, *p* = 0.98).

### Discussion

The results of Experiment 2 replicate the findings of Experiment 1 providing further support of the beneficial effects of IVI over EVI in an ecologically valid slalom based task for speed. Further, the quicker times in the IVI group were not to the detriment of accuracy, and are in line with our reasoning that IVI should help to plan the most effective line. These data also highlight that the motivational function of EVI offered by White and Hardy might be redundant in tasks where the emphasis is placed on the *control* of speed (such as driving and downhill running) rather than *generation* of speed.

The measurement of accuracy in Experiment 2 (i.e., touching the cones or not) might be perceived as rather crude. We argue that if participants chose a line that was closer to the cones at a turn, they may have more likely collided with the cone. However, this measure may not have been comprehensive enough to capture differences in accuracy of line (or trajectory) across the entire task. Consequently, a third experiment was conducted in order to explore both time-taken and accuracy, using a more comprehensive measure of accuracy.

## Experiment 3: downhill ski-slalom task

Experiment 3 explored the effects of different visual imagery perspectives on a more ecologically valid task aligned to slalom sporting performance (i.e., a downhill ski slalom task), than those used in Experiments 1 (a laboratory-based simulation slalom task) and 2 (an experimentally generated slalom task) that allowed for measures of time taken (as in Experiments 1 and 2) and accuracy (as in Experiment 2). Based on the rationale presented in Experiment 2, our *a priori* hypothesis was that IVI would produce superior performance for either time-taken or accuracy or both in comparison to EVI.

### Methods

#### Participants

A sample of 30 recreational skiers (*M* age = 24.79 years, *SD* = 4.77, 23 men, seven women) was recruited for the experiment. Although all participants could ski with their skis parallel, none had any experience of slalom skiing. All participants provided written informed consent, and ethical approval for the experiment was granted by the School's Ethics Board.

#### Task and experimental conditions

A slalom skiing task was performed twice on an outdoor artificial ski slope. The course sloped downhill at an angle of 20° and was 120 meters long with six gates. The specific course of the gates was set at an intermediate level by a qualified ski-slalom coach. Performance was assessed by the time taken to complete the course, and the accuracy of the line taken. Automatic timing gates were placed at the start and finish points of the course, and time taken to complete each trial was recorded. Each trial was videoed and an experienced ski-slalom coach blind to the nature of the experiment subsequently judged accuracy. Two criteria were used for judgments of accuracy: (a) closeness to the pole, and (b) choice of line. Each of these criteria was scored on a Likert scale from 1 (*far away from pole/very sharp change of direction*) to 10 (*just missing the pole/perfectly smooth change of direction*) and the average of these two scores was used for the accuracy measure.

Participants were allocated to an IVI, EVI, or a control group. The participants in the control group were given a series of light stretches. The participants in both imagery groups watched a brief video clip of a club level skier (not skiing the artificial ski slope) from either an internal visual or external visual perspective (the treatment matched to their imagery group perspective) and were then administered an imagery script. The imagery scripts were either from an internal visual or external visual imagery perspective depending on the group. The scripts instructed the participants to create an image of themselves skiing the course and directed them to create, in their image, the terrain, position of the poles, and the line that they should take^3^. The participants were instructed to employ imagery before each trial.

### Procedure

As with Experiments 1 and 2, imagery ability, using the VMIQ-2, was measured 1 week prior to the commencement of the experiment, with all participants achieving the specified ability criteria. Participants were then randomly assigned to groups, and to numbered bibs indicating the order in which they would each conduct the first experimental trial. Before the start of the experimental session participants were allowed a warm-up period of 20 min to ski. In three different rooms, participants were then shown the video from their respective imagery perspective group (solely to demonstrate the difference between an IVI and EVI perspective), or conducted light stretches if they were in the control group. During this time, the slalom course was erected. All participants were then allowed to walk and inspect the slalom course. This inspection lasted ~10 min. The participants then assembled in the changing room and were called individually (in bib order) to start the experimental phase. At the top of the ski slope, participants in the imagery groups were then read the imagery script and were asked to image themselves skiing the course from the respective imagery perspective. Participants in the control group conducted light stretches. In addition to this, all participants were asked to ski as quickly and as accurately as possible. Each participant then completed his/her first trial. On average, there was 30 min between the inspection of the course and the participant's first trial. The second trial took place in reverse bib order, and again was conducted on average 30 min after the first trial. Prior to performing the second trial, again at the top of the slope, participants read the imagery script themselves and were asked to image themselves skiing the course from the respective imagery perspective or complete the light stretches if in the control group. Participants were reminded to ski as quickly and as accurately as possible. For both trials, no time restrictions were placed on the participant to complete the imagery. No practice runs or discussion between participants was allowed in the changing room or while inspecting the course, and at no point during the experiment did any participant watch another participant's performance.

On completion of both trials, all participants completed a manipulation/social validation questionnaire. The questionnaire assessed; adherence to the imagery perspectives, the perceived suitability of each imagery perspective for completing the task quickly and accurately, and the experience of kinesthetic imagery for the two imagery groups. These questions were all scored on a Likert scale from 1 (*not at all*) to 10 (*greatly*). Also, participants were asked to report if they had used any other strategies to aid performance.

### Results

#### Preliminary analyses

Two participants from the control group were unable to complete both trials, leaving a sample of 28 participants. All participants in the imagery groups reported being able to adhere to their required imagery perspective and none of the participants in the control group reported using imagery to aid their performance. Data screening revealed significantly skewed and kurtotic distributions, with two outliers (one from the EVI group and one from the control group). These data points were removed and the remaining data were normally distributed. The remaining 26 participants were used for the analyses (7 control group, 10 IVI group and 9 EVI group). There were no differences between the imagery groups in whether kinesthetic imagery was experienced (*p* = 0.32, *d* = 0.55) although both groups reported experiencing kinesthetic imagery (See Table 3 for descriptive results). However, the use of kinesthetic imagery was uncorrelated with time taken (*r*_*s*_ = −0.06, *p* = 0.81) and accuracy (*r*_*s*_ = 0.03, *p* = 0.90). There were no differences in reported imagery ability (adjusted α = 0.025) across the groups for both IVI (*p* = 0.53, η^2^ = 0.05, 1-β = 0.15) and EVI ability (*p* = 0.93, η^2^ = 0.004 1-β = 0.06). The fastest of the two trials recorded was used in the data analyses.

**Table 3:** **Kinesthetic experience, time taken (seconds) and accuracy (line taken) in Experiment 3**.

**Group**	**Kinesthetic experience *M* (*SD*)**	**Time taken *M* (*SD*)**	**Accuracy *M* (*SD*)**
IVI	7.40 (1.43)	20.26 (4.10)	12.00 (1.94)
EVI	6.33 (2.45)	21.26 (2.78)	11.00 (1.73)
Control	–	23.36 (5.26)	9.86 (0.6)

#### Main analysis

Single factor ANOVAs revealed no difference between the groups for time-taken, *F*_(2, 23)_ = 1.22, *p* = 0.32, η^2^ = 0.10, 1-β = 0.24, but did reveal a significant difference for accuracy, *F*_(2, 23)_ = 3.59, *p* = 0.04, η^2^ = 0.24, 1-β = 0.61. A Tukey's follow up test indicated a significant difference between the IVI group and the control group, showing that the IVI group was more accurate than the control group (*p* = 0.04, *d* = 3.57). See Table 3 for descriptive results.

### Discussion

The results of this experiment offer some support for the hypothesis that IVI would produce superior performance than EVI in slalom based tasks. In the present data, this was demonstrated in terms of accuracy, as the IVI group was more accurate than the control group (with a large effect), whereas there was no difference between the EVI group and the control group. In terms of performance time-taken, there was no significant differences between the groups. However, the IVI group was one second quicker than the EVI group, and three seconds quicker than the control group. These differences correspond to small and moderate effect sizes of 0.30 (IVI and EVI) and 0.66 (IVI and control), respectively (cf. Cohen, 1992). Considering the time and accuracy performance results together, the findings from Experiment 3 are consistent with our theorizing that IVI may aid performance by helping to plan and execute the most accurate line. However, in isolation from the other experiments, the findings from Experiment 3 should be viewed with caution due to the low number of participants tested. The theoretical and applied implications of these findings are discussed in the General Discussion that follows.

## General discussion

The purpose of the present research was to re-examine Hardy's (1997) hypothesis that IVI will produce superior performance on slalom-based tasks compared to EVI or control conditions. Taken together, the results of the three experiments here provided support for the hypotheses set out in the present research. Specifically, there were significant performance benefits for the use of IVI compared to EVI in Experiments 1 and 2 (in terms of time taken) and significant accuracy performance benefits between IVI and control in Experiment 3 (where EVI was no better than control). Further, in Experiments 2 and 3, the differences in performance did not seem to be caused by a speed-accuracy trade-off *across* perspectives, as there were no differences between IVI and EVI for accuracy performance in Experiment 2, and no differences between IVI and the control group, for time taken in Experiment 3.

The main finding that IVI produced performance gains on these slalom-based tasks can be interpreted in line with the cognitive explanation provided by Hardy (1997), and that the neural activity in the IVI condition may be more functionally equivalent with the neural activity that occurs when performing the task in comparison to EVI (cf. Holmes and Collins, 2001; Ruby and Decety, 2001; Fourkas et al., 2006; Lorey et al., 2009). Clearly, as highlighted in the introduction, as the neural areas involved in IVI and EVI are not known, we can only propose a neural explanation. Future research that examines this issue would be particularly informative, as it would help to differentiate the neural pathways involved in the visual perspectives, and ratify the cognitive explanations that currently exist as to why visual imagery perspectives impact performance.

Importantly, the results from Experiments 2 and 3 do not provide evidence that a speed-accuracy trade-off across imagery perspectives was the cause of the performance differences (not previously controlled in White and Hardy, 1995). Specifically in Experiment 2, there was no significant differences between accuracy for the IVI and EVI conditions, and in Experiment 3, there was no significant difference in time taken between the IVI and control conditions. With this control, together these data provide the first evidence supporting the theorized benefits of IVI in slalom tasks. Indeed, these data, combined with the finding of White and Hardy (1995) and Hardy and Callow (1999) that EVI had more influence of form based performance than IVI, support the Hardy (1997) hypotheses.

An interesting additional finding in the current research was that participants reported using kinesthetic imagery regardless of imagery perspective being used, with no significant differences in the amount of reported experience between the IVI and EVI groups. These findings support the notion that kinesthetic imagery can be experienced with both visual perspectives (e.g., Glisky et al., 1996; Callow and Hardy, 2004). However, for the purpose of the present research aim, it is important to highlight that as well as no difference in the amount of kinesthetic imagery experienced between the conditions, there were also no significant correlations between kinesthetic imagery and performance in any of the experiments. As a note of interest for further investigation, previous research has reported additional performance gains for kinesthetic imagery over and above those produced by visual imagery for form-based tasks (Hardy and Callow, 1999). In the current paper, the lack of correlation between kinesthetic imagery and performance is perhaps surprising. It might be that the performance gains produced by kinesthetic imagery can only be evidenced with relatively high level performers, as measured in Hardy and Callow (1999). Thus, the lack of correlation here might be due to level of expertise of the participants on the tasks used in the present studies, and their inability to make effective or efficient use of kinesthetic imagery (cf. Hardy and Callow, 1999). In support of this contention, a recent functional magnetic resonance imaging (fMRI) study found sport experts to utilize kinesthetic imagery more efficiently (as inferred from significant blood-oxygen-level dependence response in the parahippocampus) than novices (Wei and Luo, 2010). Alternatively, the benefit of kinesthetic imagery over visual imagery might be specific to particular tasks, and based on the data here, not beneficial in slalom based tasks. We suggest that future studies should manipulate the variables of expertise, visual and kinesthetic imagery and types of task on measures of performance.

Related to the previous paragraph, we propose that it would be interesting to extend the hypotheses of Hardy (1997), and determine whether kinesthetic imagery shows specificity to particular tasks, as now demonstrated for IVI and EVI for slalom and form based tasks, respectively. Furthermore, how expertise (in both imagery ability and sport action skill) moderates the Hardy (1997) hypotheses would be of interest. In principal, the rationale provided by Hardy (1997), albeit rudimentarily, suggests that imagery modalities may prime specific actions based on similarities between the cognitive processes involved in imagining and executing specific skilled actions. Support for this explanation comes from behavioral studies coupled with neuroscience techniques (e.g., fMRI and Transcranial Magnetic Stimulation) that have examined the brain-pathways involved in imagery (cf. Moran, 2009 for a review), and the role that expertise plays in the use of imagery (Milton et al., 2008). This research generally shows that the cognitive processes differ for the different imagery modalities, and furthermore that the cognitive processes are moderated by the level of expertise of the participant.

In addition, we did not specify the angle participants should take when imagining from an EVI perspective. Although research has yet to examine whether angle of EVI affects performance, researchers (e.g., Fournier et al., 2008) have highlighted that athletes do use different angles. Manipulating angle of EVI is an obvious step for future research to consider in all imagery perspective studies (cf. Callow and Roberts, 2010). Furthermore, it would be interesting to evaluate whether moderating imaged gaze points within the internal visual imagery of a slalom task would also moderate actual performance. Finally, it is currently unknown whether supplementary sensory information such as vestibular or auditory perception moderate external and internal visual imagery in the same way. We propose that future studies manipulate additional sensory information when considering the relative roles of imagery perspective on performance.

Aside from these theoretical implications, the current experiment provides several applied implications. First, the importance of considering task characteristics when recommending to athletes which imagery perspective may be more beneficial to use is highlighted. Second, for tasks requiring an effective use of line, where a performer is required to make specific changes in direction at precise spatial locations, IVI appears to be the best imagery perspective to use to aid performance. Thus, IVI is a meaningful psychological skill for sport psychologists and coaches to develop, and for athletes to use when trying to achieve performance gains for slalom-based tasks. Third, some tasks require both form and changes in direction at precise spatial locations (e.g., a double straight-back somersault in gymnastics). With these types of task, switching between IVI and EVI might be relevant, though evidence to support the effective use of imagery switching is needed. Finally, other motor skills that do not rely so heavily on the use of form or line (such golf putting or dart throwing) might benefit equally from the use of IVI or EVI (see Roberts et al., 2010).

Certain strengths and limitations can be associated with the research presented here. Using manipulation checks in all experiments was a strength of the research, as it enabled greater experimental control (cf. Murphy and Jowdy, 1992). Employing specific imagery ability criteria, based on previous evidence (e.g., Callow et al., 2001), to accept or reject participants to the experimental phase of the studies was a strength of the research (cf. Goss et al., 1986). Further, the use of three experiments with three different tasks (that were conceptually and methodologically linked) with consistent results across the different experiments was a particular strength in relation to the general imagery literature that has traditionally relied on single studies (cf. Goginsky and Collins, 1996). Despite these strengths, there are some limitations that deserve comment. Through the use of manipulation checks to enhance experimental control, a substantial removal of participants was performed, particularly so in Experiment 2. Imagery research has previously been criticized for failing to use rigorous manipulation criteria, and so here, we felt that this approach was appropriate despite the large participant loss. Despite this removal, the remaining participant sample resulted in the hypothesized reliable effects suggesting that the conservative approach did not impact on the data. We therefore recommend that the approach is used in future related studies.

A second potential limitation of the present research was the inability to control participants' spontaneous kinesthetic imagery experiences. Although we propose that this did not influence the current findings as there were no differences in kinesthetic imagery experiences between the IVI and EVI groups and furthermore that kinesthetic imagery experience was not correlated with performance, future research may wish to explicitly control for kinesthetic imagery use. This may involve the inclusion of a kinesthetic imagery (only) condition, or it might be possible to inhibit kinesthetic imagery cognitive processes through the use of repetitive Transcranial Magnetic Stimulation (cf. Jung et al., 2008). For example, Guillot et al. (2009) found kinesthetic imagery to elicit bilateral activations of the inferior parietal lobule (BA10) as well as several motor-related regions (including the putamen, the caudate nucleus, and the cerebellar hemispheres). The application of rTMS to these brain areas may suppress kinesthetic imagery while visual imagery can still be used.

A third potential limitation relates to the measures employed. Specifically, although time-taken is a variable involved in the calculation of speed, we have not measured speed (i.e., distance/time). Further, the measure of accuracy in Studies 2 and 3 are crude. Consequently, due to possible measurement errors brought about by these limitations, the interpretations related to the speed-accuracy trade off do need to be viewed with caution. Future research plotting the spatial trajectory of the line performance, perhaps relative to a perfect line or relative to gate positions, using technology such as GPS tracking systems, would be worthwhile. With this, comparison across and within perspectives for the separate and combined effects of speed and accuracy could be made.

To conclude, the results of the present research provide evidence for the use of IVI to enhance the performance of slalom-based tasks, and enhance our knowledge in the area of imagery perspectives research.

### Conflict of interest statement

The authors declare that the research was conducted in the absence of any commercial or financial relationships that could be construed as a potential conflict of interest.

## Footnotes

^1^Standardized instructions can be obtained from the first author.

^2^Scripts can be obtained from the first author.

^3^Scripts can be obtained from the first author.
